# A rare case of breakthrough tick-borne encephalitis in early adolescence after vaccination with four doses of TBE vaccine

**DOI:** 10.1016/j.nmni.2026.101720

**Published:** 2026-02-03

**Authors:** S. Morgardt, M. Veje, T. Bergström, L. Ivarsson, M. Studahl

**Affiliations:** a Department of Infectious Diseases, NU Hospital Group, Trollhättan, Region Västra Götaland, Sweden; b Department of Infectious Diseases, Institute of Biomedicine, Sahlgrenska Academy, University of Gothenburg, Gothenburg, Sweden; c Department of Infectious Diseases, Sahlgrenska University Hospital, Gothenburg, Region Västra Götaland, Sweden; d Department of Pediatric Radiology, Queen Silvias Children's Hospital, Sahlgrenska University Hospital, Gothenburg, Region Västra Götaland, Sweden

**Keywords:** Case report, Tick-borne encephalitis, Tick-borne encephalitis virus, Breakthrough infection, Vaccination, Serology, Non-structural protein 1, Cross-reactivity, Neutralizing antibodies, Flavivirus

## Abstract

Tick-borne encephalitis (TBE) is an emerging infectious disease with effective vaccines available. However, breakthrough TBE occurs in previously vaccinated patients, a condition that is difficult to diagnose. We report a case of a previously healthy teenager who fell severely ill and was hospitalized for three months despite full TBE vaccination. Initial fever and headache progressed during the first week to encephalitis and, since autoimmune encephalitis was suspected, treatment with immunoglobulins and methylprednisolone was given. This case demonstrates the delayed response of TBEV IgM, typical for breakthrough infection, and the diagnostic value of an early IgG response to the non-structural protein 1 (NS1) in the cerebrospinal fluid. It also discusses the diagnostic challenges, the phenomenon of serological cross reactions with other flaviviruses, and the potential effect of immunomodulatory treatment in TBE. Serial MRI examinations of the brain were performed and confirmed persistent changes years after onset. The reasons for the severe outcome are discussed, but remain unclear.

## 1 Introduction

Tick-borne encephalitis (TBE) is an emerging infectious disease with increasing incidence and an extensive spread to new regions in several countries in Europe [1]. This trend is also observed in Sweden, with 380–600 cases diagnosed annually over the past five years and an expanding geographical distribution [2]. The virus causing the disease is Tick-borne encephalitis virus (TBEV), an RNA virus belonging to the group of flaviviruses causing Yellow fever, Dengue fever, Japanese encephalitis, Zika and West Nile virus infections. Cognitive sequelae are common [3] and sometimes long-lasting [4]. No antiviral or immunomodulatory treatments have been tested in clinical trials, and the latter therapies are also controversial [5]. Vaccines are accessible, but rarely implemented in national vaccination programs [6]. Vaccine breakthrough infections are reported in up to 6-7 % [7,8]. The definition of a breakthrough case varies in different studies [7,8]. The viral diagnostics of previously vaccinated patients may be challenging from several perspectives. At the presentation of symptoms, serum TBE IgG is positive as a consequence of vaccination, and the IgM response may be delayed with several weeks [9]. Recently, the analysis of non-structural protein 1 (NS1) antibodies, which are detected after prior infection but not in vaccinated patients, has emerged as a suitable and specific method to identify breakthrough TBE [[10], [11], [12]]. Serology of flaviviruses, especially IgG, may be cross-reactive [13]. Detection of viral RNA in cerebrospinal fluid (CSF) is rarely positive at the onset of neurological symptoms [14], possibly reflecting the pathogenesis of TBE, where there is a transient viremic episode weeks before neurological symptoms appear [15]. During the viremia, the virus is hypothesized to enter the central nervous system (CNS) with subsequent innate and adaptive immune responses assumed to play an important role for the CNS damage [16]. Neuroimaging has not been thoroughly investigated in TBE, but magnetic resonance imaging (MRI) shows pathological changes in about a fifth of the patients [17]. The duration of the MRI pathology is unknown, however, there are some reports of resolving changes [18,19].

## 2 Case presentation

We report a case of a previously healthy patient in early adolescence, living in an area in Sweden with increasing TBE incidence. To minimize the risk of identification, demographic details such as gender and age have been intentionally excluded. The patient had previously received four doses of TBE vaccine (FSME-IMMUN Junior), based on information extracted from the primary care electronic medical records: two primary doses seven years before disease onset, a first booster three years prior, and a second booster three months prior. The patient sustained a tick bite while staying in the western region of Sweden one week before the onset of clinical symptoms, which began with fever and headache in August (day 0). On day 5, the patient was admitted to hospital with neck stiffness and sensitivity to light, needing assistance from the parents for walking. During the examination, the patient was unable to sit or stand independently. Lumbar puncture and serum samples were drawn at admission and the result showed normal opening pressure, and pleocytosis with predominance of mononuclear cells (Table 1). Treatment was started with intravenous acyclovir, cefotaxime, ampicillin and betamethasone. An initial CT scan of the brain was normal. The first virological analyses confirmed TBEV IgG positivity and IgM negativity in serum, consistent with previous TBE vaccination (Table 1). Cerebrospinal fluid (CSF) TBEV IgM was weakly positive, which was interpreted as an unspecific reaction.

**Table 1:** Analyses performed during the acute phase and during follow-up.

	Day 5	Day 9	Day 11	Day 13	Day 16	Day 29	Day 35	Day 43	Day 64	Month 7,5	Year 1
Albumin CSF (Cut-off 225 mg/L)	340	670		400		640				150	
Lymphocytes CSF (Cut-off 4x10^6^/L)	123					76		22	4	<4	
Monocytes CSF (Cut-off 3x10^6^/L)	36	51		42		5			<3	<3	
Neutrophiles CSF (Cut-off 3x10^6^/L)	74	9		8		<3			<3	<3	
Erythrocytes CSF (Cut-off 5x10^6^/L)	12	11682		618		5				10	
Opening pressure (cm H_2_O)	15										
White blood cells serum (Reference range 4,5-13x10^9^/L)	15,8	14,6		8,4						5,7	
C-reactive protein (CRP, Cut-off 3 mg/L)	1,7	1,1		<0,5						<0,5	
TBEV IgG serum (U/ml, Cut-off 22, Euroimmun)	pos (166)		pos (>200)			pos					pos (>200)
TBEV IgM serum (U/ml, Euroimmun)	neg		pos			pos					neg
TBEV IgG CSF (U/ml, Euroimmun)	--		--			pos					
TBEV IgM CSF (OD-values, Reascan)	weakly pos		--			pos					
TBEV RNA PCR	CSF neg		urine, serum neg			serum neg					
Neutralizing antibodies TBE IgG (Cut-off <5)		20					640				
NS1 IgG serum, dilution 1/1000 (OD-values, Cut-off 0,2)	0,135		0,339		0,550	1,739		2,276			
NS1 IgM serum, dilution 1/1000 (OD-values, Cut-off 0,2)	0,088		0,296		0,718	0,490		0,755			
NS1 IgG CSF, dilution 1/10 (OD-values, Cut-off 0,2)	1,173			2,271		2,212		2,385			
NS1 IgM CSF, dilution 1/10 (OD-values, Cut-off 0,2)	0,084			2,780		2,220		1,928			
Serology for other flaviviruses (performed at Public Health Agency of Sweden)											
Dengue IgG (IF)			neg					pos			
Zika IgG (ELISA)			threshold value					pos			
Japanese encephalitis IgG (IF)			neg					pos			

Tick-borne encephalitis virus (TBEV), Cerebrospinal fluid (CSF), Polymerase chain reaction (PCR), Non-structural protein 1 (NS1), Enzyme-linked immunosorbent assay (ELISA), Immunofluorescence (IF).

**Fig. 1:**
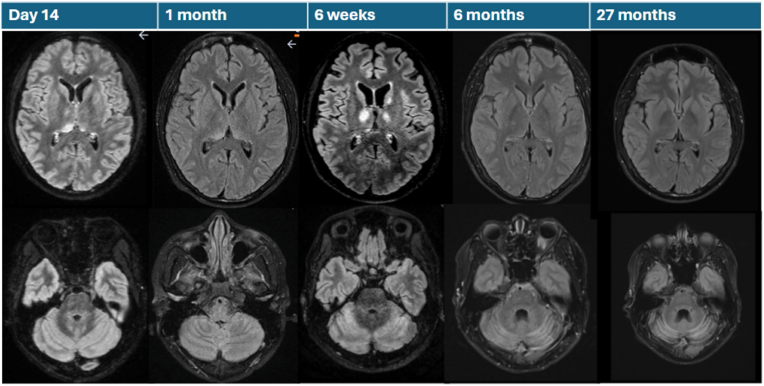
Brain MRI (Axial T2 FLAIR) series taken at different time points after onset of illness. MRI day 9 was normal (not shown). Day 14: increased signal in the posterior thalami bilaterally with a dominance on the right side, also anterior part of thalamus, and high signal in the cerebellar cortex. 1 month: partial regression seen in thalami and cerebellum. 1,5 months: renewed increased thalamic signal intensity bilaterally, both in the posterior and in the anterior part, new lesion involving the left caudate nucleus, scattered signal alterations in the cerebellum with a more pronounced oedema. 6 months: cerebellar sulcal atrophy, no oedema but increased signal in cerebellum, improvement in the thalami. 27 months: further progression of cerebellar atrophy.

After three days the patient's condition deteriorated to encephalitis with confusion, excessive motor activity, no eye contact, decreased consciousness, and bradycardia. The patient developed tonic-clonic seizures and was treated with levetiracetam. Treatment with doxycycline was also added. The first magnetic resonance imaging (MRI) of the brain was performed on day nine and was normal (image not shown). On clinical suspicion of autoimmune encephalitis, intravenous immunoglobulin (IVIG) (70 g) and methylprednisolone 1g x 1 treatments were initiated, and the patient was transferred to intensive care. Repeated examinations with electroencephalography (EEG) showed general cortical involvement as in encephalopathy. After initiation of antiepileptic therapy, no more seizures were noted. Treatment with doxycycline was stopped at day 10 when serology for *Borrelia* and *Mycoplasma* were found to be negative. The next three days the patient was circulatory and respiratory stable, however, showed no eye contact and decreased in level of consciousness with pronounced encephalopathy on EEG. On day 11, virological analyses were repeated, demonstrating serum TBEV IgM positivity and IgG increase to over 200, confirming the diagnosis of breakthrough TBE. Other etiological microbiological investigations (multiplex PCR, CSF cultures) and later on CSF autoimmune antibodies were all negative. On day 13, the patient was transferred to the University Hospital. On day 14, a renewed brain MRI (no 2) was performed and showed abnormalities and increased signals in the thalami and cerebellar cortex (Fig. 1). During the following week, there were some signs of improvement and on day 20 the patient stood up from the bed with support.

## 3 Deterioration and treatment

Starting on day 25, the patient made no contact, and the condition continued to deteriorate over the following weeks. MRI performed on day 29 (Fig. 1) showed that the previously described hyperintensity in the thalami had partially regressed. TBEV IgM was clearly positive in the CSF (Table 1). On day 31, the patient was transferred, for the second time, to the University Hospital for further diagnostics and treatment. The declined consciousness occurred after a period of clinical improvement and was suspected immunologically mediated, although autoimmune antibodies in CSF and serum repeatedly tested negative. The patient's functional decline led to a second course of immunomodulatory treatment with IVIG (8 infusions) and methylprednisolone, followed by high dose oral corticosteroids for several months, according to the BrainWorks protocol by the University of Calgary, Canada [20]. Neutralizing antibodies were analyzed in retrospect and found to be close to cut-off value on day 9, and later on at day 35, a significant titer rise confirmed the diagnosis of breakthrough TBE. These results were available before the second immunomodulatory treatment was started.

A fourth brain MRI was performed on day 43 (Fig. 1) on which renewed increased thalamic signal intensity, new lesions in the caudate nucleus and the cerebellum were found. During this period, antiepileptic medication was discontinued without new seizures occurring.

## 4 Outcome and follow-up

**Fig. 2:**
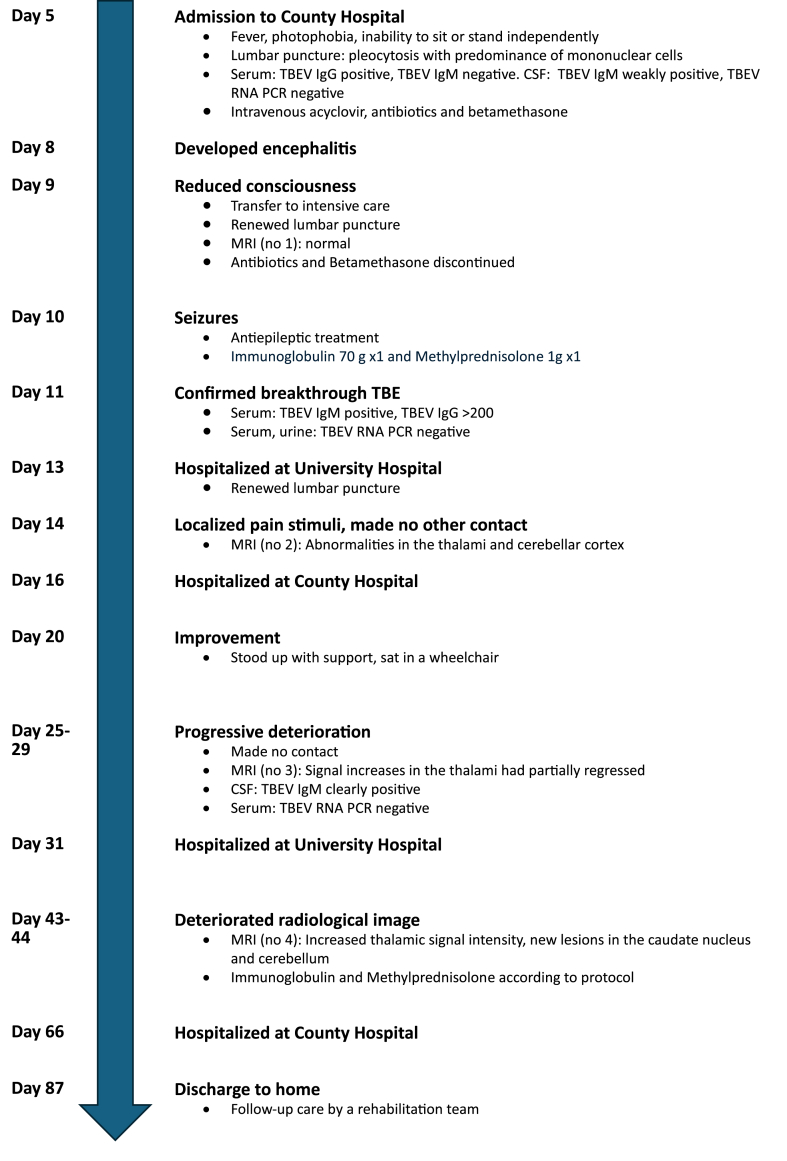
Timeline with clinical events and diagnostics from admission to discharge from hospital.

After almost three months of hospital care, the patient was discharged to home on day 87. Many motor functions were then regained but there was still near aphasia and severe brain fatigue. The patient was followed up by a physiotherapist, occupational therapist and rehabilitation services, see Fig. 2 for a timeline with clinical events and diagnostics from admission to discharge from hospital.

Over the following months, there was a gradual improvement. The fifth brain MRI after six months showed regression of changes in the thalamus and in the left caudate nucleus, with remaining signal changes in the cerebellum and some cerebellar atrophy. Seven months after onset, the patient participated in home schooling for a few hours per week, was able to speak a few words and had started to read and write but had difficulty with finger coordination. Almost two years after the TBEV infection, the patient had two seizures and was reinitiated on antiepileptic therapy. Cognitive impairments continued, including cognitive inflexibility characterized by difficulty switching tasks, problems with social communication, memory deficits, and difficulties creating structure in daily life, such as struggling with planning and maintaining routines. The sixth brain MRI performed after 27 months (Fig. 1) revealed further progression of cerebellar atrophy.

## 5 Retrospective analyses

A deepened patient history revealed that the patient was raised in Southeast Asia and moved to Sweden with relatives at the age of six. The most recent visit to the homeland was four years prior. The teenager was previously healthy and was said to have received childhood vaccinations. According to the relatives, the patient had never had Japanese encephalitis, Yellow fever or Dengue fever, and had not received vaccination against any flaviviruses other than the vaccine against tick-borne encephalitis, which was administered in Sweden. Since the patient was raised in a dengue fever endemic country, antibodies against other flaviviruses were analyzed retrospectively. At day 11, Dengue IgG and Japanese encephalitis IgG were negative and Zika virus IgG showed a threshold value. Repeated serology four weeks later detected IgG for Dengue, Zika and Japanese encephalitis virus and cross-reactivity was suspected (Table 1). In addition, the highly TBEV-specific non-structural 1 (NS1) IgG and IgM antibodies were retrospectively analyzed, and the levels were found to be elevated both in serum (day 11) and CSF (day 5) (Table 1). Whole genome sequencing, targeting 462 known immunodeficiency genes, was conducted as part of the investigation. The analysis yielded no pathogenic findings.

## 6 Discussion

The diagnostics in breakthrough TBE is challenging. Repeated serological analyses in our patient facilitated the diagnosis of breakthrough TBE since the first sample showed no TBEV IgM antibodies, but at day 11 the IgM antibodies were detected. The typical serological pattern with IgG presence in serum at presentation as a sign of previous vaccination, a delayed IgM response and intrathecal TBE antibodies are the cornerstones in virological diagnosis of breakthrough TBE. At day 29, both IgG and IgM were detected in the CSF showing comparably strong immune responses in both body fluids, a previously described phenomenon [21]. Analyzed in retrospect, our patient had IgM and IgG TBEV NS1 antibodies in serum and CSF, and the levels were found to be elevated. Especially in CSF, the response came early after onset and remained high, which illustrates the convenience of analysing NS1 antibodies in breakthrough cases as previously suggested [10,11]. Analysis of NS1 IgG in CSF during the acute phase could have enabled an earlier diagnosis. An additional advantage of TBEV NS1 is its reduced cross-reactivity with other flaviviruses compared to standard diagnostic methods [12,22]. The rise in antibody titres for other flaviviruses highlights the issues with cross-reactions among flaviviruses, which can pose a challenge in the diagnosis of TBE, especially in individuals with previous potential exposure to other flaviviruses as was the case in our patient who was brought up in Southeast Asia. Cross-reactivity means that presence of flavivirus-reactive antibodies might not be specific to TBEV but rather indicate pre-existing immunity from previous vaccinations or infections by antigenically similar flaviviruses [12]*.* Neutralizing antibody levels were observed to rise from low concentrations on day 9 to significantly elevated levels by day 35. These antibodies are not particularly useful for acute diagnostic purposes; however, TBEV-neutralizing antibodies may be applicable due to frequent serologic cross-reactivity among flaviviruses.

The reasons for the severe outcome of this case are unknown. The patient's clinical course is interpreted as monophasic, a pattern that has been associated with more severe disease manifestations [3]. The extended clinical course, characterized by intermittent neurological deterioration, may be regarded as consistent with a continuous disease course. It is conceivable that a response to the given vaccination did not develop. Unfortunately, there are no previous serum samples after vaccination but before infection available. The patient was vaccinated with two doses during the first year, followed by the first booster dose after four years, and the final booster after an additional three years. For comparison, the primary vaccination schedule in Sweden consists of three doses and is identical for previously healthy children and adults, although the injection volume differs. The first and second doses are administered 1–3 months apart, and adequate protection is expected during the subsequent tick season following these initial doses. The third dose is administered 5–12 months after the second dose and provides protection for at least three years. For individuals with marked immunosuppression or receiving immunomodulatory therapy, as well as for those aged ≥50 years at initiation of vaccination, an additional dose is recommended 2 months after the second dose to enhance vaccine-induced protection. The first booster dose is administered three years after completion of the primary series. Thereafter, booster doses are given every five years; however, in immunocompetent individuals who have received at least four doses before the age of 50, the booster interval may be extended to ten years, irrespective of previous dosing intervals [23]. The case is classified as a TBE vaccination failure according to the definition from Hansson et al., for patients diagnosed with TBE who have been vaccinated with four or more previous vaccine doses and the last vaccine dose given within <60 months [7]. It could be hypothesized that the outcome would have been different if the first booster dose (the third of a total of four doses) had been administered closer to the two priming doses. However, considering that this was a young individual without known immunodeficiency, it is unlikely that a reduced interval between the second and the third dose would have affected the immune response. This is also the conclusion of a Swedish study, which found an adequate immune response independent of the delay in administering the booster dose and irrespective of the number of previous doses of TBE vaccine [24]. The fact that TBE neutralizing IgG antibody levels at the initial assessment on day 9 were close to the cut-off rather suggests a compromised capacity to elicit an antibody response following vaccination. A recent study has raised the possibility that autoantibodies that neutralize type I interferons may underlie severe presentations of TBE. The patient, however, was never tested for these autoantibodies [25].

This case discusses the potential effect of immunomodulatory treatment, whether it may contribute to therapeutic benefits or possibly cause harm. Previous case reports have documented persistent viremia predominantly in immunosuppressed individuals, and immunomodulatory therapy may influence viral replication [26,27]. In immunocompetent individuals, with capacity to mount an inflammatory response, it is conceivable that the clinical manifestations and tissue damage may instead be driven by inflammation. Given indications that immune mediated mechanisms may contribute to CNS pathology in TBE [16,28], it is possible that the first doses of corticosteroids led to initial clinical improvement, followed by deterioration upon treatment withdrawal, as previously described [5]. When immunomodulatory treatment then was reintroduced later on, clinical improvement was once again observed. The key issue concerns the effect of treatment timing and duration on outcome. It remains uncertain whether the treatment was initiated too early, too late or terminated prematurely. There is also a possibility that antibody-dependent enhancement played a role here. Potentially, the patient may have undergone a previously asymptomatic flavivirus infection, or the effect might have been related to the immunoglobulin administered. This case also highlights the necessity of developing antiviral treatments for severe cases of TBE [29].

The patient underwent repeated MRI examinations throughout the course of the disease (Fig. 1). Increased signals were found bilaterally in the thalami, as well as in the basal ganglia and cerebellum. These findings are consistent with the typical locations where signal changes are observed in TBE, although in many cases no signal changes are detected [30,31]. Absence of findings may be attributable to early examinations, as suggested by Alkadhi and Kollias [32]. In the present case, the patient underwent several MRI scans, and it appears that pathological changes emerged with a latency of several weeks compared to the clinical presentation. An example of this is the fourth MRI performed after 1,5 months which showed renewed increased thalamic signal intensity, new lesions in the caudate nucleus and the cerebellum which correlated well with the clinical deterioration observed the weeks before. The most recent examinations demonstrated persistent cerebellar atrophy. This finding supports the presence of severe cognitive sequelae, as the cerebellum is recognized to mediate not only motor coordination but also cognitive and affective processes [33].

## 7 Key message

The IgM response to TBE in vaccinated patients is delayed, but this case demonstrated an early and strong IgG response to NS1 in the CSF, which enables rapid and specific diagnosis. Repeated MRIs in severe TBE show a few weeks' delay in the appearance of pathology compared to the clinical picture. TBE can be severe in children, even despite vaccination. There is an urgent need for trials with antiviral therapy against TBEV and additionally a more immunogenic vaccine. Optimal use and timing of immunosuppressive therapy in TBE remain unclear.

## 8 Patient consent for publication

Consent obtained from next of kin.

## CRediT authorship contribution statement

**S. Morgardt:** Writing – original draft, Visualization, Investigation, Data curation, Conceptualization. **M. Veje:** Writing – review & editing, Supervision, Investigation, Conceptualization. **T. Bergström:** Writing – review & editing, Supervision, Investigation. **L. Ivarsson:** Writing – review & editing, Resources. **M. Studahl:** Writing – review & editing, Supervision, Investigation, Conceptualization.

## Sources of funding

This work was supported by grants from the Swedish state under the ALF agreement between the Swedish Government and Country Councils (ALFGBG-1005059 and ALFGBG-1005865).

## Declaration of competing interest

Authors declare no conflicts of interest.
